# Social support needs of caregivers rearing children with severe motor and intellectual disabilities at home in Japan

**DOI:** 10.3389/fresc.2024.1280278

**Published:** 2024-01-24

**Authors:** Rie Wakimizu, Hiroshi Fujioka

**Affiliations:** ^1^ Department of Child Health and Development Nursing, Division of Health Innovation and Nursing, Institute of Medicine, University of Tsukuba, Tsukuba City, Japan; ^2^ Department of Nursing, Faculty of Health Sciences, Ibaraki Prefectural University of Health Sciences, Ibaraki, Japan

**Keywords:** children with severe motor and intellectual disabilities, family, Japan, parents, social support

## Abstract

**Background:**

In Japan, recently, the number of children with severe motor and intellectual disabilities (SMID) is steadily increasing. Caregivers such as parents and family members are struggling with how to live with their children at home and in the community after discharge.

**Objective:**

The current study aimed to explore the social support needs faced by caregivers while rearing children with SMID in order to identify effective means of social support in Japan.

**Methods:**

We conducted a cross-sectional survey of the primary caregivers of children with SMID at home through special-needs elementary, junior high, and senior high schools nationwide, using a self-administered, anonymous questionnaire to investigate the actual social support needs of the caregivers. All statements of social support need were coded using Krippendorff content analysis.

**Results:**

Questionnaire returns were obtained from 1,176 families, and the descriptions of 1,173 families were included in the analysis. The results of the analysis showed that the needs of the caregivers consisted of seven categories.

**Conclusions:**

The social support needs expressed by the caregivers are necessary findings for Japan today, both for the soft side, such as the development of local systems and regulations to support these families, and for the hard side, such as the increase and improvement of facilities and equipment.

## 1 Introduction

In Japan, SMID is defined as children who have an IQ of 35 or below and who are confined to a bed or sitting position. That is, the categorization of SMID indicates a combination of severe disabilities, both physical and mental with or without medical care. Children with SMID have been mainly selected to use medical and social services (1). The incidence of children and persons with SMID in Japan is reported to be approximately 0.2–0.4 per 1,000 (2), with an estimated number of approximately 43,000 and approximately 70% of them live at home (1, 2).

As in other countries, because of advances in medical technology and governmental policies which promotes home care, the number of children with SMID receiving home care is rapidly increasing in Japan (3). Hence, there is growing interest in the importance of family support in the home care of children with SMID.

Children with SMID require 24-h caregiving, thus, creating a physical and psychological burden for primary caregivers who are mostly mothers. They face physical and emotional strain from daily caregiving (4–6), limited opportunities to do their work or participate in social activities (7, 8), and restrictions on family time (9).

Family support in the home care of children with SMID is being implemented in Japan as disability welfare services and community life support projects based on the Comprehensive Support for Persons with Disabilities Act, which came into effect in April 2013. More specifically, there are outpatient services such as child development support, day services, and short stay (respite care services), and home-based services such as mobility support and home help services. But, the actual service provision system (e.g., the number, location, and function of service establishments) varies from region to region. The cost of equipment necessary for daily life (e.g., wheelchairs) is also partially provided. In accordance with the School Education Act, special needs schools (from elementary school to high school) have been established. Most children with SMID attend special needs schools by school bus or parent's car. In the case of children with severe medical needs, parents must be in school as well as children. This is because there are not many school nurses. In some cases, school teachers come to students’ homes to give classes (visiting classes). In this case, the number of days and hours of classes will be less than those of commuter classes. In addition, parents must also be at home.

It is important to support families rearing the children with SMID at home through multidisciplinary cooperation, with professionals in various fields such as welfare, medical care, education, and the community demonstrating their expertise in a wide range of areas, from daily living care to medical care. It has been reported that the development of these support systems has led to a situation in which caregivers are able to take a positive approach to the care of children with SMID at home (10) and it is assumed that the search for new social support contents and systems will have a significant impact on the care life of families with severely ill children at home.

Therefore, in order to examine the content and direction of further support for them, we conducted a nationwide questionnaire survey on the social support needs held by caregivers of children with SMID at home, with the aim of quantitatively collecting caregivers’ real voices to clarify the actual support needs and future support issues.

## 2 Materials and methods

### 2.1 Subjects

The subjects in the present study population consisted of primary caregivers and spouses rearing the children with SMID aged 6–18 years old at home nationwide. The term “children with SMID” is a classification based on the degree of disabilities, but not the name of a disease. A person with severe motor and intellectual disabilities (SMID) is defined as one who is bedridden or able to sit, crawl, or walk with support, and has an intelligence quotient (IQ) less than 35, according to Oshana's classification criteria (11).

### 2.2 Data collection

The subjects were recruited through all 212 special-needs schools registered with the National Federation of Parent-Teacher Associations (PTAs) for the Physically Disabled. PTA is an educational organization organized in each school unit and operated voluntarily for the purpose of conducting various activities for the growth and well-being of children and students in the community and schools with the cooperation of parents and teachers. In order to accomplish our research objective, we needed to access primary caregivers and spouses who are caring for a child with SMID living in the community, and we chose to recruit subjects via school system. First, the researcher contacted each special-needs school in Japan by telephone, explained the outline of the study, and then mailed anonymous self-administered questionnaires for the number of students concerned to those schools that had given permission for the questionnaire to be mailed. The completed questionnaires were then sealed by the subjects and submitted to each school and they were then returned to the researcher's office. The subjects were asked to answer their age, gender, and to describe freely the specific support they needed to care for children with SMID. The questionnaire we have asked is as follows. Please itemize as specifically as possible the social support you believe is necessary for the life and upbringing of the SMID child you are currently living with and caring for, reflecting on your life with the SMID child inside and outside the home, and considering the difficulties and needs in society and the future of the SMID child, you and your family. The survey period was from December 2015 to January 2016.

### 2.3 Data analysis

Descriptive statistics were performed for subject attributes, and Krippendorff's content analysis was performed for free descriptions of the specific support they needed to care for children with SMID (12). The researcher and two graduate students first carefully read each subject's description and, after understanding the content, coded one sentence or one semantic content cohesion described as necessary support for the care of severely ill children as one unit. Next, we created code clusters by focusing on the similarities and differences in the semantic content of the codes, and gave them provisional names as subcategories. A cohesion was created along the similarities and differences of the provisionally named subcategories, and a provisional category was created. In addition, the final codes, subcategories, and categories were generated by modifying the classifications and names, paying attention to the consistency, similarities, and differences in the semantic content of the codes, subcategories, and categories, and taking care not to over- or under-use the semantic content described by the subject. The researcher and two collaborators worked independently throughout the analysis, and areas of disagreement were carefully examined and corrected.

### 2.4 Ethical consideration

This study was conducted with the approval of the University of Tsukuba Medical Ethics Committee (November 2, 2015, Ethics Approval No. 1004). An explanatory letter was enclosed with the questionnaire, stating that the subject's cooperation was voluntary, that he/she would not suffer any disadvantages if he/she did not cooperate, and that his/her privacy would be protected when the results of the survey were made public. In addition, a letter to the school principals was enclosed with instructions and precautions for handling the strictly sealed questionnaires.

## 3 Results

### 3.1 Subjects

A total of 1,176 families of children enrolled in 89 out of 212 special-needs schools registered with the National Federation of PTAs for Physically Disabled Children were included in the present survey. Of these, the descriptions of a total of 1,173 primary caregivers or their spouses, in which specific details of “social support necessary for the care of children with SMID” were described, were included in the analysis. There were 704 primary caregivers and 469 spouses. The age of the subjects was 0.3% (*n* = 4) in their 20s, 20.6% (*n* = 242) in their 30s, 60.3% (*n* = 707) most in their 40s, 16.9% (*n* = 198) in their 50s, 1.4% (*n* = 16) in their 60s, and 0.5% (*n* = 6) no response. By gender, 36.0% (*n* = 422) were male, 63.2% (*n* = 741) were female, and 0.8% (*n* = 10) were non-responsive.

### 3.2 Social support needs for the care of children with SMID at home

An analysis of the “social support needs for the care of children with SMID at home” revealed that the social support needs of caregivers consisted of seven categories, 27 subcategories, and 79 codes (Table 1). In the table and in the text, categories are shown in bold, subcategories in bold italics, and codes in italics.

**Table 1:** Social support needs for the care of children with SMID at home.

	Categories are shown in bold, subcategories in bold italics, and codes in italics.	Frequency^a^
1. **Expansion of facilities and service systems** (Composed of 10 subcategories and 33 codes)		
***Increase in the number of facilities where children with medical care can be entrusted and improvement of the facility system*** (7)	*We would like more facilities to take care of children with SMID.*	468
	*We would like more medical facilities where we can leave our children with SMID without their caregivers.*	204
	*We would like more short-term care facilities for children with SMID.*	201
	*We would like to see more after-school child day care facilities for children with SMID.*	198
	*The number of children that can be accepted by each service should be expanded.*	168
	*We would like to see more facilities with knowledgeable specialists.*	124
	*We would like to see more day-care facilities after graduation.*	92
***Increase in the number of facilities with flexible deposit hours*** (3)	*We would like more facilities where I can easily leave our children with SMID.*	402
	*We would like the hours of operation of the facility to be expanded.*	289
	*We would like more short-term care facilities that accepts our children with SMID at any time.*	278
***Increase in the number of facilities where children can be left for vacations and long vacations, and improvement of facility structure*** (2)	*We would like more facilities to be available on holidays*	120
	*We would like more facilities to be available for temporary daytime care during long vacations.*	69
***Establishment of an acceptance system for facilities where you can leave your child even on urgent business*** (2)	*We want a system that allows me to leave my child with a caregiver immediately, even in times of emergency.*	56
	*We would like a system that allows me to leave my child with a caregiver in case of sudden illness of the caregiver.*	40
***Enhanced care services provided at the facility*** (2)	*We would like the variety of facilities and services available so that we can choose to use them based on our child's age, stage of development, and level of disability.*	89
	*We would like short-term residential services to be enhanced so that we can leave our children with the peace of mind that they will be cared for as if they were at home.*	88
***Mobility support*** (1)	*We need mobility support for our children with SMID.*	126
***Improved services for caregivers who care for children at home*** (8)	*We would like a service to dispatch a support person to take care of the child with SMID for a certain amount of time on my behalf (even without “caregiver's illness or other children's commitments”).*	77
	*We would like a home-based service to be dispatched for the child when the child's caregiver is unable to care for the child with SMID due to his/her own illness or other children's commitments.*	64
	*We want to expand the number of persons who can provide medical care.*	60
	*We want reliable supporters with professional medical knowledge and skills.*	58
	*We would like the expansion of housekeeping services.*	47
	*We want more bathing support services for our children with SMID.*	41
	*We want more home-visit nursing services for our children with SMID.*	25
	*We want advisors and coordinators for the home-care of the children with SMID.*	14
***Enhanced rehabilitation services*** (2)	*We would like to see more rehabilitation facilities.*	228
	*We would like the content and quality of rehabilitation services provided at the facilities to be enhanced.*	122
***Increase in the number of special-needs schools and enhancement of the medical care provision system ther***e (2)	*We would like more special needs schools.*	168
	*We would like to see more medical care provided to children in special needs schools.*	80
***Expand support for medical facilities and systems*** (4)	*We would like to be excused from accompanying us parents when our child with SMID is receiving treatment during hospitalization.*	58
	*We would like transition support from pediatrics to adult medicine.*	24
	*We would like enhance the system of medical specialists for children with SMID.*	9
	*We look forward to stronger collaboration between university hospitals and other medical institutions and local primary care physicians.*	3
2. **Enhancement of support systems** (Composed of 4 subcategories and 14 codes)		
***Establishment of laws and systems related to home care*** (4)	*We would like to see regional disparities in support systems corrected.*	88
	*We would like to see the income limit for financial aid eliminated.*	46
	*We would like to see a system in place that allows for the refurbishing of welfare equipment as the child grows.*	41
	*We would like the procedures for receiving assistance to be simplified.*	6
***Improved medical and welfare subsidies*** (6)	*We would like financial support.*	92
	*We would like the amount of benefits and allowances to be increased.*	84
	*We would like a reduction in the burden of medical expenses.*	63
	*We would like subsidies for the cost of making welfare equipment.*	41
	*We would like subsidies for the purchase of welfare vehicles.*	21
	*We would like subsidies for the purchase or remodeling of nursing homes.*	1
***Improved support attitude of administrative staff*** (1)	*We would like government officials to be friendly and approachable to our family's consultation and care situation.*	13
***Strengthen collaboration among support organizations*** (3)	*We would like cooperation between medical institutions, welfare facilities and schools to be strengthened.*	89
	*We would like to see information sharing and collaboration between local medical and administrative staff about SMID children and their families.*	82
	*We would like collaboration to be strengthened across administrative jurisdictions.*	12
3. **Usability of information, care products and services** (Composed of 3 subcategories and 6 codes)		
***Enhanced usability of information*** (2)	*We would like to have information about service resources for children with SMID in our own community.*	128
	*We would like to be informed on a daily basis about support services available to children with SMID and families in the event of a disaster.*	69
***Enhancement of usability of goods*** (1)	*We would like to improve the care products that children use on a daily basis to make them easier to use.*	45
***Enhancement of usability of consultation services*** (3)	*We would like the consultation service to provide more information and present more support.*	62
	*We would like the consultation service that specializes in providing information on service resources.*	38
	*We would like such consultation partners as peer family members or senior parents.*	30
4. **Good social connections** (Composed of 3 subcategories and 7 codes)		
***Understanding and consideration of people in society for families caring for children with SMID*** (3)	*We want people in my workplace to show understanding of my caring for children with SMID.*	106
	*We want people throughout society to show understanding of the children with SMID and their caregivers.*	57
	*We would like the priority given to people with disabilities in public places.*	42
***Social participation of families of children with SMID*** (3)	*We would like places where the children with SMID can spend time without hesitation.*	63
	*We would like places to interact with peer families.*	40
	*We would like to support and encourage the family members to participate in social activities.*	38
***Peer support*** (1)	*We would like to have more interaction with peer children and peer families.*	33
5. **Support for the future** (Composed of 2 subcategories and 4 codes)		
***Support for children after graduation from special-needs school*** (2)	*We want some places for the children with SMID to live after graduation where they can connect with peers.*	63
	*We are seeking comprehensive activity support, including physical and psychological support, for our children with SMID after graduation.*	29
***Support for aging caregivers*** (2)	*We would like to have more than one facility available when the caregivers become old and infirm and have difficulty caring for the children with SMID.*	164
	*We want a system that allows us to leave our children with SMID in the care of a facility or service with peace of mind when the caregiver becomes old and infirm and it becomes difficult for the caregiver to care for them.*	40
6. **Support for each member of the family** (Composed of 4 subcategories and 13 codes)		
***Support for caregivers*** (7)	*We would like more support to ease the burden on caregivers.*	198
	*We would like more physical support for caregivers.*	164
	*We would like more emotional support for caregivers.*	124
	*We would like more respite care.*	87
	*We would like more support to prevent caregiver's isolation.*	42
	*We would like more support for caregivers to find and keep a job.*	38
	*We would like more time with my siblings.*	13
***Support for siblings*** (2)	*We would like emotional support for siblings from non-family members.*	87
	*We would like people, place or facility to physically watch over siblings when SMID children are hospitalized.*	42
***Support for the whole family*** (1)	*We would like everyone to care about the whole family so that they are not isolated within the community where they live and the school they attend.*	7
***Understanding and cooperation of other family members*** (3)	*We would like understanding and cooperation of family members in sharing care of the children with SMID.*	162
	*We would like all family members to share household chores.*	67
	*We would like each family member other than the child with SMID to be able to live independently.*	12
7. **Improvement of the environment** (Composed of 1 subcategory and 2 codes)		
***Complete barrier-free environment*** (2)	*We would like barrier-free accessibility in every city.*	47
	*We would like more large diaper changing stations for the children with SMID in all public restrooms.*	2

^a^ The frequency of the codes emerging from data analyses.

#### 3.2.1 Expansion of facilities and service systems

This category consists of 10 subcategories and 33 codes.

In the “Increase in the number of facilities where children with medical care can be entrusted and improvement of the facility system”, there were many requests for an increase in the number of facilities and the number of people who can receive such care. A respondent wanted to use facilities with a medical care system, writing, “I want day care services with knowledgeable specialists such as nurses”.

In the “Establishment of an acceptance system for facilities where you can leave your child even on urgent business”, caregivers expressed the need for a facility where they can leave their children in the event of a sudden illness, and they wanted a facility with out-of-home support staff that can handle sudden situations that make it difficult to care for them.

In the “Enhanced care services provided at the facility”, respondents desired detailed care and services that corresponded to the child’s developmental stage and severity of illness.

In the “Improved services for caregivers who care for children at home”, Due to the background situation in Japan where only nurses and trained care workers can provide medical care such as sputum suctioning, respondents expressed a desire to “we want to expand the number of persons who can provide medical care”. Some expressed the need for advisors and coordinators to support the lives of the caregivers and the entire family indirectly through interactions with the government, in addition to supporters who provide services directly at home.

In the “Increase in the number of special-needs schools and enhancement of the medical care provision system there”, one of the reasons why “caregivers want more medical care at special-needs schools” is that special-needs schools currently require caregivers to accompany their children in order to provide medical care, which places a burden on caregivers and prevents them from playing an active role in society.

In the “Expand support for medical facilities and systems”, the needs for smooth transitional medical care as the child grows up was noted, “We would like transition support from pediatrics to adult medicine”. The caregivers also expressed their wish for “stronger cooperation among medical institutions”, such as between departments of the same hospital and between primary and core hospitals in the region.

#### 3.2.2 Enhancement of support systems

This category consists of 4 subcategories and 14 codes.

In the “Establishment of laws and systems related to home care”, there was a need to “correct regional disparities in support systems”, such as available service resources and care products provided. They also asked for a review of laws and systems, saying, “We would like to see the income limit for financial aid eliminated”, and “We would like to see a system in place that allows for the refurbishing of welfare equipment as the child grows”. They also stated “We would like the procedures for receiving assistance to be simplified” and felt burdened by the time and effort required when applying for and renewing support.

In the “Improved support attitude of administrative staff”, they expressed their wish that government officials would show a more attentive and approachable attitude to their families’ stories.

In the “Strengthen collaboration among support organizations”, the respondents expressed a need to strengthen cooperation between professionals and institutions involved with children and their families by sharing information.

#### 3.2.3 Usability of information, care products and services

In the “Enhanced usability of information”, they wanted information on the services available to their children in the event of a disaster, fearing that the services they use daily would be unavailable.

In the “Enhancement of usability of consultation services”, they wanted not only a full counseling service but also “someone to talk to” about their problems from the bottom of their hearts, and they wanted to connect with other people.

#### 3.2.4 Good social connections

In the “Understanding and consideration of people in society for families caring for children with SMID”, the caregivers sought understanding in three areas for the disabled child and his/her family: understanding of the caregivers as a whole, as in “We want people in my workplace to show understanding of my caring for children with SMID”, understanding of the caregivers as in “We want people throughout society to show understanding of the children with SMID and their caregivers”, and understanding of the entertainment facilities, including movie theaters, as in “We would like the priority given to people with disabilities in public places”.

In the “Social participation of families of children with SMID”, there was a desire for a variety of “places for exchange”, such as places that can be used by the whole family and where children can interact with each other, regardless of whether they have disabilities or not.

In the “Peer support”, the opinion was expressed that they would like to incorporate consultation and study sessions with senior caregivers raising children with SMID.

#### 3.2.5 Support for the future

In the “Support for children after graduation from special-needs school”, families were looking for a place for their children to connect with society in place of school, as expressed in the need for “We want some places for the children with SMID to live after graduation where they can connect with peers”.

In the “Support for aging caregivers”, they expected to receive general support to take care of their children until the end of their lives, writing that they needed facilities and systems that would allow them to leave their children in peace when they became elderly or passed away.

#### 3.2.6 Support for each member of the family

In the “Support for aging caregivers”, many of the needs were related to the care burden of the caregivers, such as “We would like more support to ease the burden on caregivers” and “We would like more respite care”. On the other hand, the primary caregivers wanted to balance care and work, as in “We would like more support for caregivers to find and keep a job”. The caregivers raising siblings with children with SMID were described as “We would like more time with my siblings”.

In the “Support for Siblings”, the respondents also wanted both emotional and physical support for their siblings. The needs identified were “emotional support for siblings from non-family members” and “support for physically watching over siblings when SMID children are hospitalized”.

#### 3.2.7 Improvement of the environment

In the “Complete barrier-free environment”, the needs for barrier-free access throughout the city, including transportation, roads, elevators, and private facilities, were described. Specific needs such as “We would like more large diaper changing stations for the children with SMID in all public restrooms” were also noted.

## 4 Discussion

### 4.1 Expansion of facilities and service systems

Four of the ten subcategories indicated a need for an increase in the number of facilities, and caregivers complained of a lack of facilities and the number of people who can take care of severely disabled children. While Japan's neonatal and infant mortality rates continue to decline year after year, and the number of lives that can be saved despite serious disabilities is increasing, the number of medical-type residential facilities for severely disabled children has remained unchanged over the past several years (13). It is necessary to confirm once again whether sufficient resources are reaching families in need.

In 2018, the System for Publication of Information on Disability Welfare Services began in Japan, enabling caregivers to search and browse the Internet for services of “medical-type residential facilities for children with disabilities” and “medical-type child development support” (14). Such Internet services are expected to make it easier for caregivers of children with SMID to select medical care and support services that meet their needs.

On the other hand, caregivers of SMID children were perplexed by the differences in medical care provided by nurses at schools and hospitals, and were anxious and dissatisfied with the medical care provided at schools. Another issue is the current situation in which teachers must acquire a great deal of specialized knowledge, such as understanding the disabilities of children with SMID and how to use medical equipment to provide medical care, within the limited training time available (15). It is necessary to enhance the content of training based on the needs of caregivers regarding medical care at schools and to focus on educating teachers who receive certification.

Regarding medical facilities and systems, the survey revealed the need and necessity for transitional care from pediatrics to adult care and from hospital to community, as well as the accompanying collaboration among medical institutions. It was thought that these needs were raised as advances in medical care and the diffusion and spread of medical care have enabled severely ill children to continue to live in the community.

### 4.2 Enhancement of support systems

One of the reasons for the need for “We would like to see the income limit for financial aid eliminated” was the conditions under which financial assistance is provided. The special child allowance currently in effect in Japan is based on the income of one primary earner, so even if the household income is lower than other families, if the income of the primary earner, the caregiver, exceeds the limit, the allowance is not paid (14). The number of dual-earner households in Japan is increasing, and families raising seriously ill children are no exception. It is necessary to adapt the current system to current social conditions, for example, by making the total income of the household a condition for receiving the allowance.

In the “Strengthen collaboration among support organizations”, cooperation among medical care, welfare, school, community, and government is required. If there is good communication and coordination among these agencies, a cooperative system can be established and appropriate intervention can be made at an early stage. It is important to build a system to support children with SMID and their families by understanding the characteristics of each agency and sharing the current situation and issues as needed through liaison exchange meetings, etc., so that the support agencies can build a system to support children with SMID and their families.

### 4.3 Usability of information, care products, and services

The need to know about service resources stood out. In particular, the need for information on services available to children in the event of a disaster was identified, indicating that caregivers are concerned about whether they will be able to get the support they need in the event of a disaster. It is important to be prepared for disasters on a regular basis, such as having an external battery for the ventilator and carrying a help card with emergency contact information and medical information (16). Prior surveys in Japan have revealed that even families raising children with disabilities have low awareness of the Disaster Evacuation Support Plan for Persons Requiring Assistance (17). It was considered necessary for administrative officials in each municipality to encourage such families to register on the list of persons requiring assistance, participate in local evacuation drills, and build face-to-face relationships with local residents in order to develop their mutual assistance capabilities. Furthermore, service providers should work closely with local government disaster management departments to help families caring for children with SMID acquire new service users and continue to use services in case of disasters.

### 4.4 Good social connections

The caregiver expressed the need for understanding in the workplace. Caregivers of children with disabilities may have to limit their working hours to pick up and drop off their children or leave work early due to sudden changes in their children's physical condition, and in order for them to continue working, it is necessary for people in the workplace to understand their medical care situation. Activities are needed to expand people's understanding of the reality and burden of rehabilitation.

The caregivers wanted to have “peer exchange”, including consultation with senior foster caregivers and study sessions. Sharing experiences and information through peer exchange is a learning experience for caregivers, and can help them clarify their own intentions regarding their children's caring or rehabilitation and encourage them to make decisions regarding the use of new services. Peer exchange is thought to prevent their social isolation and improve their well-being and quality of life (18).

### 4.5 Support for the future

The need for “We want some places for the children with SMID to live after graduation where they can connect with peers” suggested that caregivers want SMID children to have connections with others, not simply a place where they can spend time. Since school is a valuable place for children to socialize with peers, SMID children who have difficulty finding work may become isolated from society after school. Therefore, it is necessary to secure in advance a place for them in the future where they can maintain their social skills. However, since they are unable to work and require assistance in daily living, it is difficult for them to become independent. However, this study highlighted the fact that caregivers are anxious about the future because they are aware that they will not be able to care for their SMID children when their aging caregivers themselves need care or pass away. Previous study has shown that they fear their absence may result in worse care and support for their child and consequentially a diminished quality of life (QoL) (19). Based on the above two points, in addition to securing a place where SMID children can live, it is considered necessary to develop a support system and build a society in which children can be cared for safely even in the absence of their caregivers.

### 4.6 Support for each member of the family

The primary caregivers wanted support not only for the care of their children, but also for their own employment. The reasons for wanting to work varied from the family's financial situation to a desire for personal contact with society. It was thought that active intervention/support was needed for the social aspect as well as the physical and mental health of the primary caregivers, rather than simply stating that it was inevitable that the caregivers could not work because of the heavy burden of caring for the children.

This study confirmed previous research showing that caregivers of children with special healthcare needs feel conflicted about not being able to fully interact with their siblings (20, 21). The need for “We would like emotional support for siblings from non-family members” was considered to be caused by the fear that the parent's own neglect of the siblings as a parent might cause frustration among the siblings.

It is necessary for supporters and society as a whole to promote communication between parents and siblings and to support the healthy growth of siblings together with caregivers by considering not only SMID children and caregivers, but also siblings as one of the targets of support.

### 4.7 Improvement of the environment

Most children with SMID require some form of assistance with toileting and use diapers. Currently in Japan, the installation of diaper changing stations is recommended in public transportation and facility restrooms, and some local governments require diaper changing stations in large department stores. However, most diaper changing stations are for infants and may be too small & narrow to use for grown children with SMID. Therefore, public restroom designers need to assume users with various characteristics and pursue more universal designs, but it is difficult to make immediate improvements because it takes money and time to improve the facility environment. However, it would be a good idea for the concerned caregivers and local governments to work together in the future on the search for accessible toilet locations and available diaper changing stations and other devices.

### 4.8 Limitations and future research

The authors aimed to explore the social support needs faced by caregivers while rearing the child with SMID in order to identify effective means of social support in Japan. This paper is clearly relevant to healthcare practice. However, our main concern regarding this manuscript is the outdated knowledge it presents. The study was conducted from December 2015 to January 2016, before the COVID pandemic to which we generally assign a significant impact, and in the past eight years, it might have raised of important changes in the needs and supports for families and individuals with disabilities and the regional healthcare system might have undergone minor improvements, which could potentially impact the perspectives and social support needs of caregivers rearing children with SMID at home. A new data collection might then be very interesting both in describing changes as related to actions undertaken in the meantime and at the same time give space for reflections on new and challenging needs.

Despite the limitations described earlier, the results of this survey provided us with the real voices of caregivers raising severely ill children at home and revealed a wide range of needs, from those directed at individual support organizations to those directed at society. Based on the results of this survey, it is necessary to consider specific support for the children and their families, and to support them so that they and their families can lead healthy lives in the community.

## 5 Conclusion

In this study, using Krippendorff content analysis, we were able to obtain the real voices of caregivers raising children with SMID at home and clarify the reality of a wide range of needs, from those directed at individual support agencies to those directed at society as a whole.

## Data Availability

The original contributions presented in the study are included in the article/Supplementary Material, further inquiries can be directed to the corresponding author.
